# Machine Learning–Based Short-Term Mortality Prediction Models for Patients With Cancer Using Electronic Health Record Data: Systematic Review and Critical Appraisal

**DOI:** 10.2196/33182

**Published:** 2022-03-14

**Authors:** Sheng-Chieh Lu, Cai Xu, Chandler H Nguyen, Yimin Geng, André Pfob, Chris Sidey-Gibbons

**Affiliations:** 1 Department of Symptom Research The University of Texas MD Anderson Cancer Center Houston, TX United States; 2 McGovern Medical School University of Texas Health Science Center Houston, TX United States; 3 Research Medical Library The University of Texas MD Anderson Cancer Center Houston, TX United States; 4 Department of Obstetrics and Gynecology Heidelberg University Hospital Heidelberg Germany

**Keywords:** machine learning, cancer mortality, artificial intelligence, clinical prediction models, end-of-life care

## Abstract

**Background:**

In the United States, national guidelines suggest that aggressive cancer care should be avoided in the final months of life. However, guideline compliance currently requires clinicians to make judgments based on their experience as to when a patient is nearing the end of their life. Machine learning (ML) algorithms may facilitate improved end-of-life care provision for patients with cancer by identifying patients at risk of short-term mortality.

**Objective:**

This study aims to summarize the evidence for applying ML in ≤1-year cancer mortality prediction to assist with the transition to end-of-life care for patients with cancer.

**Methods:**

We searched MEDLINE, Embase, Scopus, Web of Science, and IEEE to identify relevant articles. We included studies describing ML algorithms predicting ≤1-year mortality in patients of oncology. We used the prediction model risk of bias assessment tool to assess the quality of the included studies.

**Results:**

We included 15 articles involving 110,058 patients in the final synthesis. Of the 15 studies, 12 (80%) had a high or unclear risk of bias. The model performance was good: the area under the receiver operating characteristic curve ranged from 0.72 to 0.92. We identified common issues leading to biased models, including using a single performance metric, incomplete reporting of or inappropriate modeling practice, and small sample size.

**Conclusions:**

We found encouraging signs of ML performance in predicting short-term cancer mortality. Nevertheless, no included ML algorithms are suitable for clinical practice at the current stage because of the high risk of bias and uncertainty regarding real-world performance. Further research is needed to develop ML models using the modern standards of algorithm development and reporting.

## Introduction

### Background

Cancer therapies, including chemotherapy, immunotherapy, radiation, and surgery, aim to cure and reduce the risk of recurrence in early-stage disease and improve survival and quality of life for late-stage disease. However, cancer therapy is invariably associated with negative effects, including toxicity, comorbidities, financial burden, and social disruption. There is growing recognition that therapies are sometimes started too late, and many patients die while receiving active therapy [1-3]. For instance, a systematic review summarized that the percentage of patients with lung cancer receiving aggressive treatments during the last month of their life ranged from 6.4% to >50% [4]. Another retrospective comparison study revealed that the proportion of patients with gynecologic cancer undergoing chemotherapy or invasive procedures in their last 3 months was significantly higher in 2011 to 2015 than in 2006 to 2010 [5]. Research has shown that the aggressiveness of care at the end of life in patients with advanced cancers is associated with extra costs and a reduction in the quality of life for patients and their families [4,6].

In the United States, national guidelines state that gold standard cancer care should avoid the provision of aggressive care in the final months of life [7]. Avoiding aggressive care at the end of life currently requires clinicians to make judgments based on their experience as to when a patient is nearing the end of their life [8]. Research has shown that these decisions are difficult to make because of a lack of scientific, objective evidence to support the clinicians’ judgment in palliative or related discussion initiation [2,9,10]. Thus, a decision support tool enabling the early identification of patients of oncology who may not benefit from aggressive care is needed to support better palliative care management and reduce clinicians’ burden [2].

In recent years, there have been substantial changes in both the type and quantity of patient data collected using electronic health records (EHR) and the sophistication and availability of the techniques used to learn the complex patterns within that data. By learning these patterns, it is possible to make predictions for individual patients’ future health states [11]. The process of creating accurate predictions from evident patterns in past data is referred to as machine learning (ML), a branch of artificial intelligence research [12]. There has been growing enthusiasm for the development of ML algorithms to guide clinical problems. Using ML to create robust, individualized predictions of clinical outcomes, such as the risk of short-term mortality [13,14], may improve care by allowing clinical teams to adjust care plans in anticipation of a forecasted event. Such predictions have been shown to be acceptable for use in clinical practice [15] and may one day become a fundamental aspect of clinical practice.

ML applications have been developed to support mortality predictions for a variety of populations, including but not limited to patients with traumatic brain injury, COVID-19 disease of 2019, and cancers, as well as patients admitted to emergency departments and intensive care units. These applications have consistently demonstrated promising performances across studies [16-19]. Researchers have also applied ML techniques to create tools supporting various clinical tasks involved in the care of patients of oncology, with most applications focusing on the prediction of cancer susceptibility, recurrence, treatment response, and survival [14,19,20]. However, the performance of ML applications in supporting mortality predictions for patients of oncology has not yet been systematically examined and synthesized.

In addition, as the popularity of ML in clinical medicine has risen, so too has the realization that applying complex algorithms to big data sets does not in itself result in high-quality models [11,21]. For example, subtle temporal-regional nuances in data can cause models to learn relationships that are not repeated over time and space. This can lead to poor future performance and misleading predictions [22]. Algorithms may also learn to replicate human biases in data and, as a result, could produce predictions that negatively affect disadvantaged groups [23,24]. Recent commentary has drawn attention to various issues in the transparency, performance, and reproducibility of ML tools [25-27]. A comparison of 511 scientific papers describing the development of ML algorithms found that, in terms of reproducibility, ML for health care compared poorly to other fields [28]. Issues of algorithmic fairness and performance are especially pertinent when predicting patient mortality. If done correctly, these predictions could help patients and their families receive gold standard care at the end of life; if done incorrectly, there is a risk of causing unnecessary harm and distress at a deeply sensitive time.

Another aspect of mortality affecting the algorithm performance is its rare occurrence in most populations. There are known issues that are commonly encountered when trying to predict events from data sets in which there are far fewer events than nonevents, which is known as class imbalance. One such issue is known as the *accuracy paradox*—the case in which an ML algorithm presents with high accuracy but a failure to identify occurrences of the rare outcome it was tasked to predict [29,30]. During the model training process, many algorithms seek to maximize their accuracy across the entire data set. In the case of a data set in which only 10% of patients experienced a rare outcome—as is often the case with data sets containing mortality—an algorithm could achieve an apparently excellent accuracy of 0.90 by simply predicting that every patient would live. The resulting algorithm would be clinically useless on account of its failure to identify patients who are at risk of dying. If handled incorrectly, the class imbalance problem can lead algorithms to prioritize the predictions of the majority class. For this reason, it is especially important to evaluate multiple performance metrics when assessing algorithms that predict rare events.

### Objective

The purpose of this systematic review is to critically evaluate the current evidence to (1) summarize ML-based model performance in predicting ≤1-year mortality for patients with cancer, (2) evaluate the practice and reporting of ML modeling, and (3) provide suggestions to guide future work in the area. In this study, we seek to evaluate models identifying patients with cancer who are near the end of their life and may benefit from end-of-life care to facilitate the better provision of care. As the definitions of aggressive care at the end of life vary from initiation of chemotherapy or invasive procedures or admission to the emergency department or intensive care unit within 14 days to 6 months [1,4,5], we focused on ≤1-year mortality of patients with cancer to ensure that we include all ML models that have the potential to reduce the aggressiveness of care and support the better provision of palliative care for cancer populations.

## Methods

### Overview

We conducted this systematic review following the Joanna Briggs Institute guidelines for systematic reviews [31]. To facilitate reproducible reporting, we present our results following the PRISMA (Preferred Reporting Items for Systematic Reviews and Meta-Analyses) statement [32]. This review was prospectively registered in PROSPERO (International Prospective Register of Systematic Reviews; PROSPERO ID: CRD42021246233). The protocol for this review has not been published.

### Search Strategy

We searched Ovid MEDLINE, Ovid Embase, Clarivate Analytics Web of Science, Elsevier Scopus, and IEEE Xplore databases from the date of inception to October 2020. The following concepts were searched using subject headings keywords as needed: *cancer*, *tumor*, *oncology*, *machine learning*, *artificial intelligence*, *performance* *metrics*, *mortality*, *cancer death*, *survival rate*, and *prognosis*. The terms were combined using AND/OR Boolean statements. A full list of search terms along with a complete search strategy for each database used is provided in Multimedia Appendix 1. In addition, we reviewed the reference lists of each included study for relevant studies.

### Study Selection

A total of 2 team members screened all the titles and abstracts of the articles identified in the search for studies. A senior ML researcher (CSG) resolved the discrepancies between the 2 reviewers. We then examined the full text of the remaining articles using the same approach but resolved disagreements via consensus. Studies were included if they (1) developed or validated ML-based models predicting ≤1-year mortality for patients of oncology, (2) made predictions using EHR data, (3) reported model performance, and (4) were original research published through a peer-reviewed process in English. We excluded studies if they (1) focused on risk factor investigation; (2) implemented existing models; (3) were not specific to patients with cancer; (4) used only image, genomic, clinical trial, or publicly available data; (5) predicted long-term (>1 year) mortality or survival probability; (6) created survival stratification using unsupervised ML approaches; and (7) were not peer-reviewed full papers. We defined short-term mortality as death happening within ≤1 year after receiving cancer diagnostics or certain treatments for this review.

### Critical Appraisal

We evaluated the risk of bias (ROB) of each included study using the prediction model ROB assessment tool [33]. A total of 2 reviewers independently conducted the assessment for all the included studies and resolved conflicts by consensus.

### Data Extraction and Synthesis

For data extraction, we developed a spreadsheet based on the items in the transparent reporting of a multivariable prediction model for individual prognosis or diagnosis (TRIPOD) [34] through iterative discussions. A total of 4 reviewers independently extracted information about sampling, data sources, predictive and outcome variables, modeling and evaluation approaches, model performance, and model interpretations using the spreadsheet from the included studies, with each study extracted by 2 reviewers. Discrepancies were discussed among all reviewers to reach a consensus. The collected data items are available in Multimedia Appendix 2 [35]. To summarize the evidence, we grouped the studies using TRIPOD’s classification for prediction model studies (Textbox 1) and summarized the data narratively and descriptively by group. To estimate the performance of each ML algorithm, we averaged the area under the receiver operating characteristic curve (AUROC) for each type of ML algorithm across the included studies and estimated SE for 95% CI calculation using the averaged AUROC and pooled validation sample size for each type of ML algorithm. In addition, we conducted a sensitivity analysis to assess the impact of studies that were outliers either on the basis of their sample size or their risk of bias.

**Types of prediction model studies.**

**Study type and definition**

**Type 1a**

Studies develop prediction model or models and evaluate model performance using the same data used for model development.

**Type 1b**

Studies develop prediction model or models and evaluate the model or models using the same data used for model development with resampling techniques (eg, bootstrapping and cross-validation) to avoid an optimistic performance estimate.

**Type 2a**

Studies randomly split data into two subsets: one for model development and another for model performance estimate.

**Type 2b**

Studies nonrandomly split data into two subsets: one for model development and another for model performance estimate. The splitting rule can be by institute, location, and time.

**Type 3**

Studies develop and evaluate prediction model or models using 2 different data sets (eg, from different studies).

**Type 4**

Studies evaluate existing prediction models with new data sets not used in model development.

Note: The types of prediction model studies were summarized from Collins et al [34].

## Results

### Summary of Included Studies

**Figure 1:**
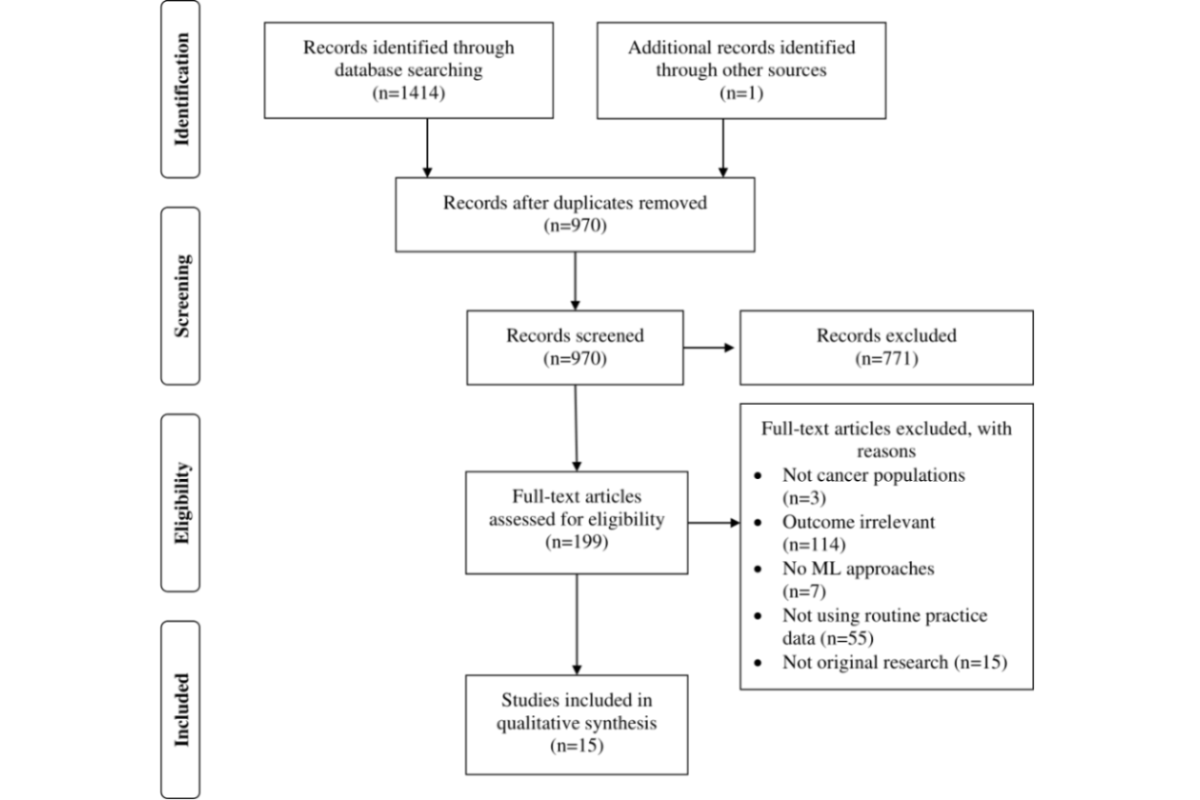
PRISMA (Preferred Reporting Item for Systematic Reviews and Meta-Analyses) flowchart diagram for the study selection process. ML: machine learning.

Our search resulted in 970 unduplicated references, of which we excluded 771 (79.5%) articles because of various reasons, such as no ML involvement, not using EHR data, or no patient with cancer involvement, based on the title and abstract screen. After the full-text review, we included 1.5% (15/970) of articles involving a total of 110,058 patients with cancer (Figure 1). We have provided a detailed record of the selection process in Multimedia Appendix 3 [36,37].

We present a characteristic summary of the included articles in Table 1 [36-49]. Of the 15 included articles, 13 (87%) were model development and internal validations, and 2 (13%) were external validations of existing models. The median sample size was 783 (range 173-26,946), with a median of 21 predictors considered (range 9-5390). The target populations of the 15 articles included 5 (33%) with all types of cancer, 3 (20%) with spinal metastatic diseases, 2 (13%) with liver cancer, and 1 (7%) each with gastric cancer, colon and rectum cancer, stomach cancer, lung cancer, and bladder cancer. Several algorithms have been examined in many studies. The most commonly used ML algorithms were artificial neural networks (8/15, 53%). Other algorithms included gradient-boosted trees (4/15, 27%), decision trees (4/15, 27%), regularized logistic regression (LR; 4/15, 27%), stochastic gradient boosting (2/15, 13%), naive Bayes classifier (1/15, 7%), Bayes point machine (1/15, 7%), and random forest (RF; 1/15, 7%). Of the 15 studies, 2 (13%) tested their models in their training data sets by resampling (type 1b), 9 (60%) examined their models using randomly split holdout internal validation data sets (type 2a), 2 (13%) examined with nonrandomly split holdout validation data sets (type 2b), and 2 (13%) validated existing models using external data sets (type 4). The frequent candidate predictors were demographic (12/15, 80%), clinicopathologic (12/15, 80%), tumor entity (7/15, 47%), laboratory (7/15, 47%), comorbidity (5/15, 33%), and prior treatment information (5/15, 33%). The event of interest varied across the studies, with 47% (7/15) for 1-year mortality, 33% (5/15) for 180-day mortality, 13% (2/15) for 90-day mortality, and 7% (1/15) for 30-day mortality.

**Table 1:** Characteristics of the included studies (N=15).

Type of cancer and study			Country		Study type		Treatment		Sample size							Algorithms			Input features (total number of features)			Outcome
									Training		Testing		Validating									
**All cancer**																						
	Sena et al [38]	Brazil		1b		All		543		N/A^a^		N/A		DT^b^, ANN^c^, and NB^d^			Comorbidity and PRO^e^ for physical and mental status assessments (9)			180-day death		
	Parikh et al [39]	United States		2a		All		18,567		7958		N/A		GBT^f^ and RF^g^			Demographic, clinicopathologic, laboratory, comorbidity, and electrocardiogram data (599)			180-day death		
	Manz et al [37]	United States		4		All		N/A		N/A		24,582		GBT			Same as Parikh et al [39]			180-day death		
	Bertsimas et al [50]	United States		2a		All		14,427		9556		N/A		DT, regularized LR^h^, and GBT			Demographic, clinicopathologic, gene mutations, prior treatment, comorbidity, use of health care resources, vital signs, and laboratory data (401)			180-day death		
	Elfiky et al [43]	United States		2b		All		17,832		9114		N/A		GBT			Demographic, clinicopathologic, prescription, comorbidity, laboratory, vital sign, and use of health care resources data and physician notes (5390)			180-day death		
**Non–small cell lung cancer**																						
	Hanai et al [44]	Japan		2b		Curative resection		125		48		N/A		ANN			Demographic, clinicopathologic, and tumor entity data (17)			1-year death		
**Gastric cancer**																						
	Nilsaz-Dezfouli et al [45]	Iran		1b		Surgery		452		N/A		N/A		ANN			Demographic, clinicopathologic, tumor entity, and prior treatment (20)			1-year death		
**Colon and rectum cancer**																						
	Arostegui et al [46]	Spain		2a		Curative or palliative surgery		981		964		N/A		DT and regularized LR			Demographic, clinicopathologic, tumor entity, comorbidity, ASA^i^ prior treatment, laboratory, operational data, postoperational complication, and use of health care resources data (32)			1-year death		
**Stomach cancer**																						
	Biglarian et al [47]	Iran		2a		Surgery		300		136		N/A		ANN			Demographic, clinicopathologic, and symptom data (NR^j^)			1-year death		
**Bladder cancer**																						
	Klén et al [48]	Turkey		2a		Radical cystectomy		733		366		N/A		Regularized LR			Demographic, clinicopathologic, ASA, comorbidity, laboratory, prior treatment, tomography, and operational data (NR)			90-day death		
**Hepatocellular carcinoma**																						
	Chiu et al [49]	Taiwan		2a		Liver resection		347		87		N/A		ANN			Demographic, clinicopathologic, tumor entity, comorbidity, ASA, laboratory, operational, and postoperational data (21)			1-year death		
	Zhang et al [40]	China		2a		Liver transplant		230		60		N/A		ANN			Donor demographic data and recipient laboratory, clinicopathologic, and image data (14)			1-year death		
**Spinal metastatic**																						
	Karhade et al [41]	United States		2a		Surgery		1432		358		N/A		ANN, SVM^k^, DT, and BPM^l^			Demographic, clinicopathologic, tumor entity, ASA, laboratory, and operational data (23)			30-day death		
	Karhade et al [42]	United States		2a		Surgery		587		145		N/A		SGB^m^, RF, ANN, SVM, and regularized LR			Demographic, clinicopathologic, tumor entity, laboratory, operational, ECOG^n^, ASIA^o^, and prior treatment data (29)			90-day death		
	Karhade et al [36]	United States		4		Curative surgery		N/A		N/A		176		SGB			ECOG, demographic, clinicopathologic, tumor entity, laboratory, prior treatment, and ASIA data (23)			1-year death		

^a^N/A: not applicable.

^b^DT: decision tree.

^c^ANN: artificial neural network.

^d^NB: naive Bayes.

^e^PRO: patient-reported outcome.

^f^GBT: gradient-boosted tree.

^g^RF: random forest.

^h^LR: logistic regression.

^i^ASA: American Sociological Association.

^j^NR: not reported.

^k^SVM: support vector machine.

^l^BPM: Bayes point machine.

^m^SGB: stochastic gradient boosting.

^n^ECOG: Eastern Cooperative Oncology Group.

^o^ASIA: American Spinal Injury Association.

### ROB Evaluation

**Figure 2:**
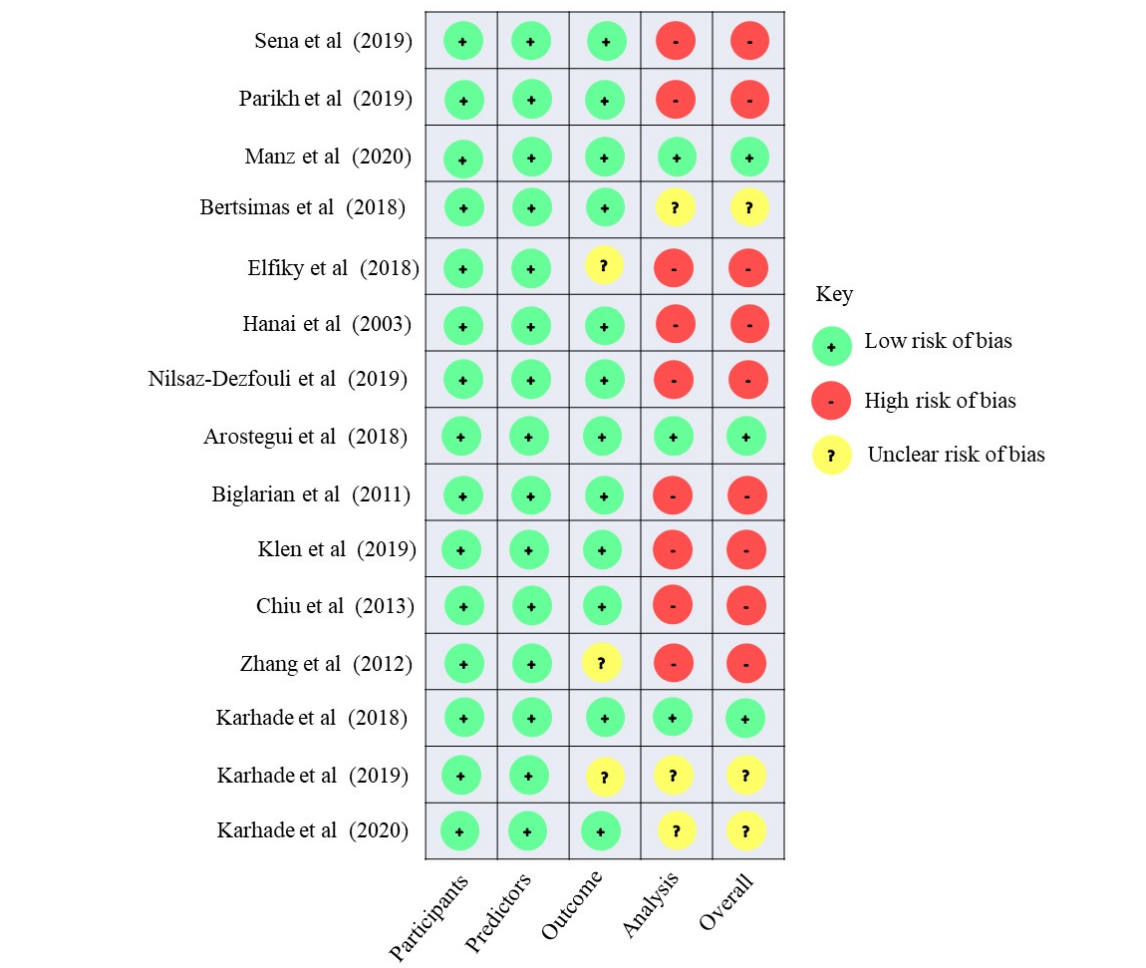
Risk of bias assessment for the included studies. Risk of bias assessment result for each included study using prediction model risk of bias assessment tool [15,35-49].

Of the 15 studies, 12 (80%) were deemed to have a high or unclear ROB. The analysis domain was the major source of bias (Figure 2). Of the 12 model development studies, 8 (67%) provided insufficient or no information on data preprocessing and model optimization (tuning) methods. Approximately 33% (5/15) of studies did not report how they addressed missing data, and 13% (2/15) potentially introduced selection bias by excluding patients with missing data. All studies clearly defined their study populations and data sources, although none justified their sample size. Predictors and outcomes of interest were also well-defined in all studies, except for 20% (3/15) of studies that did not specify their outcome measure definition and whether the definition was consistently used.

### Model Performance

We summarize the performance of the best models from the type 2, 3, and 4 studies (12/15,80%) in Table 2. We excluded 1 type 2b study as the authors did not report their performance results in a holdout validation set. Model performance across the studies ranged from acceptable to good, based on AUROC ranging from 0.72 to 0.92. Approximately 40% (6/15) of studies reported only the AUROC values, therefore, leaving some uncertainty about model performance in correctly identifying patients at risk of short-term mortality. Other performance metrics were less reported and were sometimes indicative of poor performance. Studies reported median accuracy 0.91 (range 0.86-0.96; 2/15, 13%), sensitivity 0.85 (range 0.27-0.91; 4/15, 27%), specificity 0.90 (0.50-0.99; 5/15, 33%), as well as the positive predictive value (PPV) and the negative predictive value of 0.52 (range 0.45-0.83; 4/15, 27%) and 0.92 (range 0.86-0.97; 2/15, 13%), respectively.

**Figure 3:**
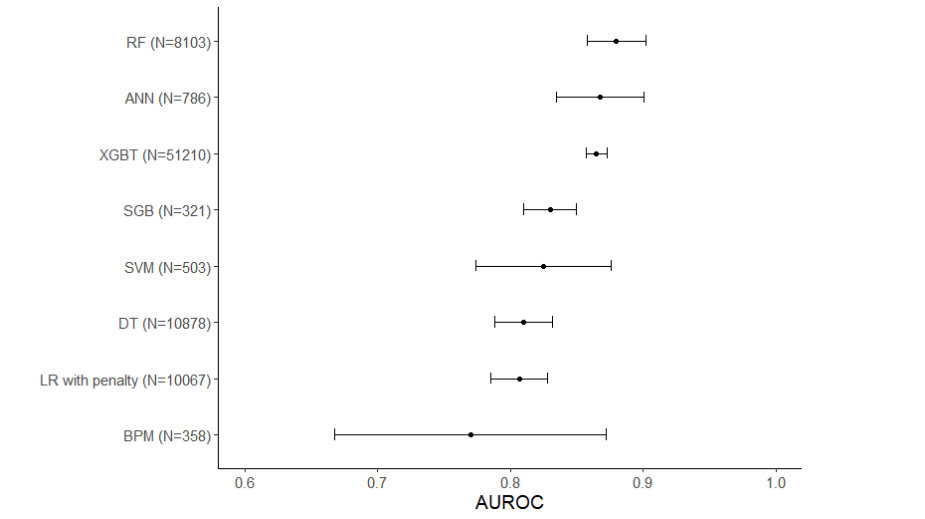
Pooled AUROC by machine learning (ML) algorithm. ANN: artificial neural network; AUROC: area under the receiver operating characteristic curve; BPM: Bayes point machine; DT: decision tree; GBT: gradient-boosted tree; LR: logistic regression; RF: random forest; SGB: stochastic gradient boosting; SVM: support vector machine.

Among the ML algorithms examined, all algorithms were similarly performed, with RF slightly better than the other algorithms (Figure 3). Approximately 33% (5/15) of studies compared their ML algorithms with statistical models [39,47,48,50,51]. Differences in AUROC between the ML and statistical models ranged from 0.01 to 0.11, with one of the studies reporting a significant difference (Table 2).

**Table 2:** Predicting performance for the best model for each study in a holdout internal or external validation data set (N=12).

Type of cancer and study		Outcome	Training sample	Validation sample	Mortality rate (%)	Algorithm	AUROC^a^	Accuracy	Sensitivity	Specificity	PPV^b^	NPV^c^	Calibration	Benchmark, model (Δ AUROC)	
**All cancer**															
	Manz et al [37]	180-day death	N/A^d^	24,582	4.2	GBT^e^	0.89	—^f^	0.27	0.99	0.45	0.97	Well-fit	—	
	Parikh et al [39]	180-day death	18,567	7958	4.0	RF^g^	0.87	0.96	—	0.99	0.51	—	Well-fit at the low-risk group	LR^h^ (0.01)	
	Bertsimas et al [50]	180-day death	14,427	9556	5.6	GBT	0.87	0.87	.60	—	0.53	—	—	LR (0.11)	
	Elfiky et al [43]	180-day death	17,832	9114	18.4	GBT	0.83	—	—	—	—	—	Well-fit	—	
**Gastrointestinal cancer**															
	Arostegui et al [46]	1-year death	981	964	5.1	DT^i^	0.84	—	—	—	—	—	Well-fit	—	
	Biglarian et al [47]	1-year death	300	136	37.5	ANN^j^	0.92	—	0.80	0.85	—	—	—	CPH^k^ (0.04)^l^	
**Patients with bladder cancer**															
	Klén et al [48]	90-day death	733	366	4.4	Regularized LR	0.72	—	—	—	—	—	—	ACCI^m^ univariate model (0.05)	
**Patients with liver cancer**															
	Chiu et al [49]	1-year death	347	87	17	ANN	0.88	—	0.89	0.50	—	—	—	LR (0.08)	
	Zhang et al [40]	1-year death	230	60	23.9	ANN	0.91	—	0.91	0.90	0.83	0.86	—	—	
**Patients with spinal metastasis**															
	Karhade et al [41]	30-day death	1432	358	8.5	BPM^n^	0.78	—	—	—	—	—	Well-fit	—	
	Karhade et al [42]	1-year death	586	145	54.3	SGB^o^	0.89	—	—	—	—	—	Well-fit	—	
	Karhade et al [36]	1-year death	N/A	176	56.2	SGB	0.77	—	—	—	—	—	Fairly well-fit	—	

^a^AUROC: area under the receiver operating characteristic curve.

^b^PPV: positive predictive value.

^c^NPV: negative predictive value.

^d^N/A: not applicable.

^e^GBT: gradient-boosted tree.

^f^No data available

^g^RF: random forest.

^h^LR: logistic regression.

^i^DT: decision tree.

^j^ANN: artificial neural network.

^k^CPH: Cox proportional hazard.

^l^Significant at the α level defined by the study.

^m^ACCI: adjusted Charlson comorbidity index.

^n^BPM: Bayes point machine

^o^SGB: stochastic gradient boosting.

### Model Development and Evaluation Processes

Most articles (11/15, 73%) did not report how their training data were preprocessed (Table 3). Authors of 27% (4/15) of articles reported their methods for preparing numeric variables, with 75% (3/4) using normalization, 25% (1/4) using standardization, and 25% (1/4) using discretization. Approximately 13% (2/15) of articles used one-hot encoding for their categorical variables. Various techniques were used to address missing data, including constant value imputation (3/15, 20%), multiple imputation (3/15, 20%), complete cases only (2/15, 13%), probabilistic imputation (1/15, 7%), and the optimal impute algorithm (1/15, 7%).

Of the 13 model development studies, 9 (69%) reported their approaches for feature selection. The approaches, including 3 model-based variable importance, between-variable correlation, zero variance, univariate Cox proportional hazard, forward stepwise selection algorithm, recursive feature selection, and parameter-increasing method, were used alone or in combination. Concerning hyperparameter selection, 33% (5/15) reported their methods to determine hyperparameters, with 60% (3/5) using grid search and 2 (40%) using the default values of the modeling software. Finally, 47% (7/15) used various resampling approaches to ensure the generalizability of their models. The N-fold cross-validation approach was the primary strategy. Varying fold numbers were used, such as 10 (3/15, 20%), 5 (2/15, 13%), 4 (1/15, 7%), 3 repeats 10 (1/15, 7%), and 5 repeats 5-fold (1/15, 7%). One of the studies used the bootstrapping method. Approximately 27% (4/15) of studies did not report whether resampling was performed.

Of the 15 studies, 12 (80%) used variable importance plots to interpret their models, 3 (20%) included decision tree rules, and 2 (13%) included coefficients to explain their models in terms of prediction generation. Other model interpretation approaches, including local interpretable model-agnostic explanations and partial dependence plots, were used in 7% (1/15) of studies.

**Table 3:** The Model development processes and evaluations used in the included studies.

Type and study		Data preprocessing				Model optimization				Interpretation
		Numeric variables	Categorical variables	Missing data	Feature selection		Hyperparameter value selection	Generalizability consideration		
**Type 1b**										
	Sena et al [38]	Normalization	N/A^a^	NR^b^	None		Software default	10-fold CV^c^	VI^d^	
	Nilsaz-Dezfouli et al [45]	NR	NR	NR	VI		Grid search	5×5-fold CV	VI	
**Type 2a**										
	Parikh et al [39]	NR	NR	Constant value imputation	Zero variance and between-variable correlation		Grid search	5-fold CV	VI and coefficient	
	Klén et al [48]	NR	NR	Complete cases only	LASSO^e^ LR^f^		NR	NR	VI	
	Karhade et al [42]	NR	NR	missForest multiple imputation	RF^g^		NR	3×10-fold CV	VI, PDP^h^, and LIME^i^	
	Karhade et al [41]	NR	NR	Multiple imputation	Recursive feature selection		NR	10-fold CV	NR	
	Arostegui et al [46]	Discretization	One-hot encoding	Constant value imputation	RF variable importance		Software default	Bootstrapping	VI and decision tree rules	
	Bertsimas et al [50]	NR	NR	Optimal impute algorithm	None		NR	NR	VI and decision tree rules	
	Chiu et al [49]	NR	NR	Complete cases only	Univariate Cox proportional hazard model		NR	NR	VI	
	Zhang et al [40]	Normalization	One-hot encoding	NR	Forward stepwise selection algorithm		NR	10-fold CV	VI	
	Biglarian et al [47]	NR	NR	NR	None		NR	NR	NR	
**Type 2b**										
	Elfiky et al [43]	NR	NR	Probabilistic imputation	None		Grid search	4-fold CV	VI	
	Hanai et al [44]	Standardization	NR	NR	Between-variable correlation and PIM^j^		NR	5-fold CV	VI	
**Type 4**										
	Manz et al [37]	NR	NR	Constant value imputation	N/A		N/A	N/A	VI and coefficient	
	Karhade et al [36]	NR	NR	missForest multiple imputation	N/A		N/A	N/A	NR	

^a^N/A: not applicable.

^b^NR: not reported.

^c^CV: cross-validation.

^d^VI: variable importance.

^e^LASSO: least absolute shrinkage and selection operator.

^f^LR: logistic regression.

^g^RF: random forest.

^h^PDP: partial dependence plot.

^i^LIME: local interpretable model-agnostic explanation.

^j^PIM: parameter-increasing method.

### Solutions for Class Imbalance

All included studies reported that the mortality rate of their samples experienced some degree of class imbalance (Table 3). The median mortality rate was 20.0% (range 4%-56.2%), with 2.8 deaths in training samples per candidate predictor (range 0.5-12.3) in training samples. A type 1 study discussed the potential disadvantage of the issue and used a downsampling approach to handle imbalanced data. No information was provided on how the downsampling approach was conducted and its effectiveness on model performance in an unseen data set.

### Sensitivity Analysis

Owing to the small number of included studies, we conducted a sensitivity analysis by including 1 study per research group to avoid the disproportionate effects of studies from a single group on our model performance and modeling practice evaluation. We observed similar issues concerning model development and evaluation practice after removing the studies by Manz et al [37] and Karhade et al [36,41]. For model performance, all algorithms still demonstrated good performance, with a median AUROC of 0.88 ranging from 0.81 to 0.89 (Multimedia Appendix 4 [36,37,41]). We detected changes in AUROC for all algorithms except RF and regularized LR (ranging from −0.008 to 0.065). Stochastic gradient boosting and support vector machine algorithms had the greatest changes in AUROC (ΔAUROC=0.06 and 0.065, respectively). However, the performance of these models in the sensitivity analysis may not be reliable as both algorithms were examined in the same study using a small sample (n=145).

## Discussion

### Principal Findings

Mortality prediction is a sensitive topic that, if done correctly, could assist with the provision of appropriate end-of-life care for patients with cancer. ML-based models have been developed to support the prediction; however, the current evidence has not yet been systematically examined. To fill this gap, we performed a systematic review evaluating 15 studies to summarize the evidence quality and the performance of ML-based models predicting short-term mortality for the identification of patients with cancer who may benefit from palliative care. Our findings suggest that the algorithms appeared to have promising overall discriminatory performance with respect to AUROC values, consistent with previous studies summarizing the performance of ML-based models supporting mortality predictions for other populations [16-19]. However, the results must be interpreted with caution because of the high ROB across the studies, as well as some evidence of the selective reporting of important performance metrics such as sensitivity and PPV, supporting previous studies reporting poor adherence to TRIPOD reporting items in ML studies [52]. We identified several common issues that could lead to biased models and misleading model performance estimates in the methods used to develop and evaluate the algorithms. The issues included the use of a single performance metric, incomplete reporting of or inappropriate data preprocessing and modeling, and small sample size. Further research is needed to establish a guideline for ML modeling, evaluation, and reporting to enhance the evidence quality in this area.

We found that the AUROC was predominantly used as the primary metric for model selection. Other performance metrics have been less discussed. However, the AUROC provides less information for determining whether the model is clinically beneficial, as it equally weighs sensitivity and specificity [53,54]. For instance, Manz et al [37] reported a model predicting 180-day mortality for patients with cancer with an AUROC of 0.89, showing the superior performance of the model [37]. However, their model demonstrated a low sensitivity of 0.27, indicating poor performance in identifying individuals at high risk of 180-day death. In practice, whether to stress sensitivity or specificity depends on the model’s purpose. In the case of rare event prediction, we believe that sensitivity will usually be prioritized. Therefore, we strongly suggest that future studies report multiple discrimination metrics, including sensitivity, specificity, PPV, negative predictive value, F1 score, and the area under the precision–recall curve, to allow for a comprehensive evaluation [53-55].

We found no clear difference in performance between general and cancer-specific ML models for short-term mortality predictions (AUROC 0.87 for general models vs 0.86 for cancer-specific models). This finding aligns with a study reporting no performance benefit of disease-specific ML models over general ML models for hospital readmission predictions [56]. However, among the 15 included studies, 10 (67%) examined ML performance in short-term mortality for only a few types of cancer, which resulted in the ML in most cancer types remaining unexplored and compromising the comparison. In fact, a few disease-specific models examined in this review demonstrated exceptional performance and have the potential to provide disease-specific information to better guide clinical practice [40,47]. As such, we recommend that more research test ML models using various oncology-specific patient cohorts to predict short-term mortality to enable a full understanding of whether disease-specific ML models can bring advantages over limitations, such as higher development and implementation cost.

Only 33% (5/15) of the included studies compared their model with a traditional statistical model, such as univariate or multivariate LR [39,47,48,50,51]. Of the 15 studies, 1 (7%) reported that ML models were statistically more accurate, although all studies reported a superior AUROC of their ML models compared with statistical predictive models. This finding supports previous studies that reported that the performance benefit of ML over conventional modeling approaches is unclear at the current stage [57]. Thus, although we argue that the capacity of ML algorithms in dealing with nonlinear, high-dimensional data could benefit clinical practice by identifying additional risk factors for intervening to improve patient outcomes beyond predictive performance, we encourage researchers to benchmark their ML models against conventional approaches to highlight the performance benefit of ML.

Our review suggests that the sample size consideration is missing for ML studies in the field, which is consistent with a previous review [58]. In fact, none of the included studies justified the appropriateness of their sample size, given the number of candidate predictors used in model development. Simulation studies have suggested that most ML modeling approaches require >200 data points related to the outcome per candidate predictor to reach a stable performance and mitigate optimistic models [59]. Unfortunately, none of the included studies met this criterion. Thus, we recommend that future studies justify the appropriateness of their sample size and use feature selection and dimensional reduction techniques before modeling to reduce the number of candidate predictors if a small sample is inevitably used.

Most studies used imbalanced data sets without additional procedures to address the issue, such as over- or downsampling. The effects of class-imbalanced data sets are unclear as sensitivity was often unreported and widely varied when it was reported. A study used a downsampling technique to balance their data set [38]. However, the authors did not report their model performance in a holdout validation data set. Thus, the effectiveness of this approach is unknown. Moreover, the effectiveness of other approaches, such as the synthetic minority oversampling technique [60], remains unexamined in this context. Further research is needed to examine whether these approaches can further improve the performance of ML models in predicting cancer mortality.

Most ML models predicting short-term cancer mortality were reported without intuitive interpretations of the prediction processes. It has been well-documented that ML acceptance by the larger medical community is limited because of the limited interpretability of ML-based models [53]. Despite the widespread use of variable importance analysis to reveal essential factors for the models in the included studies, it is unknown how the models used the factors to generate the predictions [61]. As the field progresses, global and local model interpretation approaches have been developed to explain ML models intuitively and visually at a data set and instance level [61]. The inclusion of these analyses to provide an intuitive model explanation may not only gain medical professionals’ trust but also provide information guiding individualized care plans and future investigations [62]. Therefore, we highly recommend that future studies *unbox* their models using various explanation analyses in addition to model performance.

### Limitations

This review has several limitations. First, we did not quantitatively synthesize the model performance because of the clinical and methodological heterogeneity of the included studies. We believe that a meta-analysis of the model performance would provide clear evidence but should be conducted with enough homogeneous studies [63]. Second, the ROB of the studies may be inappropriately estimated because of the use of the prediction model ROB assessment tool checklist, which was developed for appraising predictive modeling studies using multivariable analysis. Some items may not apply, or additional items may be needed because of the differences in terminology, theoretical foundations, and procedures between ML-based and regression-based studies. Finally, the results of this review may be affected by reporting bias as we did not consider studies published outside of scientific journals or in non-English languages. Furthermore, our results could be compromised by the small number of included studies and the inclusion of studies by the same group (eg, 3 studies from Karhade et al [36,41,42]). However, we observed similar issues with model development and performance in our sensitivity analysis, suggesting that our evaluation likely reflects the current evidence in the literature. Despite these limitations, this review provides an overview of ML-based model performance in predicting short-term cancer mortality and leads to recommendations concerning model development and reporting.

### Conclusions

In conclusion, we found signs of encouraging performance but also highlighted several issues concerning the way algorithms were trained, evaluated, and reported in the current literature. The overall ROB was high, and there was substantial uncertainty regarding the development and performance of the models in the real world because of incomplete reporting. Although some models are potentially clinically beneficial, we must conclude that none of the included studies produced an ML model that we considered suitable for clinical practice to support palliative care initiation and provision. We encourage further efforts to develop safe and effective ML models using modern standards of development and reporting.

## Appendix

Multimedia Appendix 1 Search strategy for all reference databases used.

Multimedia Appendix 2 Data collection tool.

Multimedia Appendix 3 Detailed record for the study selection process.

Multimedia Appendix 4 Comparison of area under the receiver operating characteristic curve for each machine learning algorithm between the full and sensitivity analyses.

## Abbreviations

AUROC: area under the receiver operating characteristic curve
EHR: electronic health record
LR: logistic regression
ML: machine learning
PPV: positive predictive value
PRISMA: Preferred Reporting Items for Systematic Reviews and Meta-Analyses
PROSPERO: International Prospective Register of Systematic Reviews
RF: random forest
ROB: risk of bias
TRIPOD: the transparent reporting of a multivariable prediction model for individual prognosis or diagnosis
