# Supplementary material for: Machine Learning–Based Short-Term Mortality Prediction Models for Patients With Cancer Using Electronic Health Record Data: Systematic Review and Critical Appraisal
Source: JMIR Med Inform. 2022 Mar 14;10(3):e33182. doi: 10.2196/33182 (PMC8961346; doi:10.2196/33182)
Supplement: Multimedia Appendix 1 [file medinform_v10i3e33182_app1.docx]

**Multimedia Appendix 1**

Supplementary Table 1. Search strategy for all reference databases used

| # | Searches |
| --- | --- |
| **MEDLINE** | |
| 1 | exp Neoplasms/ |
| 2 | exp Medical Oncology/ |
| 3 | (cancer* or carcinom* or tumor* or tumour* or neoplas* or malignan* or myeloma* or leuk?emia* or lymphoma* or sarcoma* or melanoma* or oncolog*).ti,ab,kf. |
| 4 | Oncology Nursing/ |
| 5 | or/1-4 [Cancer or oncology] |
| 6 | limit 5 to english language |
| 7 | (animals not (humans and animals)).sh. |
| 8 | 6 not 7 |
| 9 | (mice or mouse or murine or rat or rats or rodent or cells or "in vitro" or "cell line").ti. |
| 10 | 8 not 9 |
| 11 | exp Artificial Intelligence/ |
| 12 | ("machine learning" or "artificial intelligence" or "expert system*" or "deep learning" or "natural language processing" or "neural network*" or "fuzzy logic*").ti,ab,kf. |
| 13 | or/11-12 |
| 14 | 10 and 13 [Machine learning in oncology] |
| 15 | Mortality/ |
| 16 | "Cause of Death"/ |
| 17 | Hospital Mortality/ |
| 18 | fatal outcome/ |
| 19 | Survival Rate/ |
| 20 | (mortalit* or fatalit*).ti,ab,kf. |
| 21 | (cancer adj3 (death* or dying or died)).ti,ab,kf. |
| 22 | or/15-21 [(cancer) survival] |
| 23 | 14 and 22 [Machine learning and cancer mortality] |
| 24 | exp Prognosis/ |
| 25 | predict*.ti,ab,kf. |
| 26 | (prognostic or prognosis).ti,ab,kf. |
| 27 | (disease adj5 course).ti,ab,kf. |
| 28 | or/24-27 [prediction or prognosis] |
| 29 | 23 and 28 [Machine learning for predicting cancer mortality/death] |
| 30 | ((prognostic or prognosis) adj2 (value or model* or predict*)).ti,ab,kf. |
| 31 | (predict* adj5 (death or mortality* or fatalit*)).ti,ab,kf. |
| 32 | (predict* adj2 (model* or system* or validat* or performance*)).ti,ab,kf. |
| 33 | exp "Sensitivity and Specificity"/ |
| 34 | exp Area Under Curve/ |
| 35 | (Sensitivity or Specificity or accuracy or "ROC Curve*" or "Area Under Curve" or PPV or NPV or "predictive value").ti,ab,kf. |
| 36 | (recall or "F-measure*" or "F-score*" or "F1-score*" or "F1-measure*").ti,ab,kf. |
| 37 | or/30-36 [performance measurement of ML/NLP model] |
| 38 | 29 and 37 |
| **EMBASE** | |
| 1 | exp malignant neoplasm/ |
| 2 | (cancer* or carcinom* or tumor* or tumour* or neoplas* or malignan* or myeloma* or leuk?emia* or lymphoma* or sarcoma* or melanoma* or oncolog*).ti,ab. |
| 3 | 1 or 2 [cancer] |
| 4 | limit 3 to english language |
| 5 | Human/ |
| 6 | Nonhuman/ or ANIMAL/ or Animal Experiment/ |
| 7 | 6 not 5 |
| 8 | 4 not 7 |
| 9 | (mice or mouse or murine or rat or rats or rodent or cells or "in vitro" or "cell line").ti. |
| 10 | 8 not 9 |
| 11 | exp artificial intelligence/ |
| 12 | exp machine learning/ |
| 13 | expert system/ |
| 14 | natural language processing/ |
| 15 | ("machine learning" or "artificial intelligence" or "expert system*" or "deep learning" or "natural language processing" or "neural network*").ti,ab. |
| 16 | or/11-15 [machine learning] |
| 17 | 10 and 16 [Machine learning in oncology] |
| 18 | cancer mortality/ |
| 19 | (cancer adj3 (mortalit* or fatalit* or death* or died)).ti,ab,kw. |
| 20 | 18 or 19 [cancer mortality] |
| 21 | 17 and 20 [Machine learning and cancer mortality] |
| 22 | cancer prognosis/ |
| 23 | (prognostic or prognosis).ti. |
| 24 | exp "prediction and forecasting"/ |
| 25 | predict*.ti,ab,kw. |
| 26 | or/22-25 [prediction or prognosis] |
| 27 | 21 and 26 [Machine learning for predicting cancer mortality/death] |
| 28 | ((prognostic or prognosis) adj2 (value or predict*)).ti. |
| 29 | (cancer adj3 mortalit*).ti,ab. and exp *"prediction and forecasting"/ |
| 30 | (predict* adj5 (death* or mortality* or fatalit*)).ti,ab,kw. |
| 31 | (predict* adj2 (model* or system* or validat* or performance*)).ti,ab,kw. |
| 32 | exp "sensitivity and specificity"/ |
| 33 | exp area under the curve/ |
| 34 | receiver operating characteristic/ |
| 35 | exp recall/ |
| 36 | accuracy/ |
| 37 | predictive value/ |
| 38 | (Sensitivity or Specificity or accuracy or "ROC Curve*" or "Area Under Curve" or PPV or NPV or "predictive value").ti,ab,kw. |
| 39 | (recall or "F-measure*" or "F-score*" or "F1-score*" or "F1-measure*").ti,ab,kw. |
| 40 | or/28-39 [performance measurement of ML/NLP model] |
| 41 | 27 and 40 |
| 42 | conference abstract.pt. |
| 43 | 41 and 42 |
| **SCOPUS** | |
| 1 | TITLE ( cancer* OR carcinom* OR tumor* OR tumour* OR neoplas* OR malignan* OR metasta* OR myeloma* OR leukemia* OR leukaemia* OR lymphoma* OR sarcoma* OR melanoma* OR oncolog* ) ) |
| 2 | TITLE-ABS-KEY ( cancer OR tumor OR tumour OR neoplasm ) |
| 3 | 1 or 2 |
| 4 | TITLE-ABS-KEY ( "machine learning" OR "artificial intelligence" OR "expert system*" OR "deep learning" OR "natural language processing" OR "neural network*" ) |
| 5 | TITLE-ABS-KEY ( mortalit* OR fatalit* OR ( cancer AND w3 AND death* ) ) |
| 6 | TITLE-ABS-KEY ( predict* ) |
| 7 | TITLE-ABS-KEY ( ( predict* W/30 ( death* OR mortalit* OR died OR dying ) ) ) |
| 8 | 3 and 4 and 5 and 6 and 7 |
| 9 | 8 LIMIT-TO ( LANGUAGE , "English" ) |
| **Web of Science**  Indexes=SCI-EXPANDED, SSCI, A&HCI, CPCI-S, CPCI-SSH, ESCI Timespan=1900-2020 | |
| 1 | TOPIC: ((cancer* or carcinom* or tumor* or tumour* or neoplas* or malignan* or metasta* or myeloma* or leukemia* or leukaemia* or lymphoma* or sarcoma* or melanoma* or oncolog*) ) |
| 2 | TS=(("machine learning" or "artificial intelligence" or "expert system*" or "deep learning" or "natural language processing" or "fuzzy logic*" or "neural network*" )) |
| 3 | #2 AND #1 |
| 4 | TS=(mortalit* or fatalit*) |
| 5 | TS=(cancer NEAR/3 death*) |
| 6 | #5 OR #4 |
| 7 | #6 AND #3 |
| 8 | TS=(predict*) |
| 9 | #8 AND #7 |
| 10 | TS=(predict* NEAR/30 (death* or mortalit* or died or dying) ) |
| 11 | #10 AND #9 |
| **IEEE Xplore** | |
| 1 | (((((((No Keywords Specified))) AND ((All Metadata:mortalit*))) OR ((All Metadata:cancer NEAR/3 death*))) AND ((No Keywords Specified))) AND ((All Metadata:cancer AND All Metadata:predict* ))) AND ((All Metadata:machine learning OR artificial intelligence OR natural language processing OR neural network* OR deep learning)) |
