# Supplementary material for: Machine Learning–Based Short-Term Mortality Prediction Models for Patients With Cancer Using Electronic Health Record Data: Systematic Review and Critical Appraisal
Source: JMIR Med Inform. 2022 Mar 14;10(3):e33182. doi: 10.2196/33182 (PMC8961346; doi:10.2196/33182)
Supplement: Multimedia Appendix 2 [file medinform_v10i3e33182_app2.docx]

**Multimedia Appendix 2**

Supplementary Table 2. Itemized data collection tool

| Category | Item | Description | Example |
| --- | --- | --- | --- |
| Basic information | Author | The first author of the article | Oh, et al.[1] |
|  | Title | Title of the included reference | Prediction of Overall Survival and Novel Classification of Patients with Gastric Cancer Using the Survival Recurrent Network |
|  | Year | Year the article was published | 2018 |
|  | Journal | Journal publishing the article | Annals of Surgical Oncology |
|  | Purpose | Study aim(s) of the article | To develop a new survival prediction model using an ANN with a large number of gastric cancer patients who had undergone radical gastrectomy |
|  | Tumor entities | Type of tumor or cancer | Gastric cancer |
|  | Tumor stages | Tumor stages | Stage IIA–IV |
|  | Treatment | Treatments that participant had received | Radical gastrectomy |
| Method | Overall design | Study design for data collection, and model development, validation, and/or refinement | Retrospective cohort study |
|  | ML algorithm(s) | Machine learning algorithm(s) used to develop the prediction model(s) | Survival recurrent  network (SRN) |
|  | Data | Information about the dataset used to train and test the models. The information may include where, when, and how the data were collected, as well as who collected the data. | Data collected from medical records between January 2007 to June 2010 |
|  | Sample size - total | Total number of participants in the analysis | 1243 |
|  | Sample size - Training | Total number of participants in training dataset | 995 |
|  | Sample size - testing | Total number of participants in testing dataset | 248 |
|  | Sample size - validation | Total number of participants in the dataset for external validation | N/A |
|  | Sample size – Sample size calculation | Do the authors discuss whether their sample size for development /validation is appropriate? (Yes/No) | No |
|  | Data splitting | How data were split for training, testing, and validation. (Random/Non-random) | Random |
|  | Splitting ratio | The ratio used to split dataset | 4:1 |
|  | Data preparation | All data preprocessing performed on the dataset. Common processes include log transformation, scale standardization, missing data imputation, oversampling/undersampling, removing correlated and near-zero variance features, one-hot encoding, and dimensionality reduction | Scale standardization, missing data imputation, one-hot encoding |
|  | Candidate covariates | Candidate covariates, variables, and features included in the analysis | age, sex, extent of resection, tumor location and size (maximum and minimum), resection margin (proximal and distal), histologic types, Lauren classification, depth of invasion, total number of dissected lymph nodes, number of metastatic lymph nodes, lymph node metastases (N stage), distant metastases, pathologic stage, lymphatic involvement, venous involvement, perineural involvement and, postoperative  chemotherapy |
|  | Total number of candidate covariates | Total number of covariates used in the study | 17 |
|  | Feature selection | Methods to determine candidate covariates (e.g., previous literature, data availability, univariate/multivariate analysis, genetic algorithm, Boruta, variable importance from ML algorithms, etc.) | Previous literature |
|  | Primary outcomes | Primary events/outcomes that the prediction model will predict (x-year overall survival /distant recurrence-free survival etc.). | 5-year mortality |
|  | Class distribution | Class distribution of the outcome in training dataset or entire dataset (Survive vs Die) | 9:1 |
|  | Methods for addressing class imbalance | If class imbalance exists, what method authors used to address it (e.g., none, undersampling, oversampling, etc.) | None |
|  | Model optimization-cross-resampling approach | What resampling approach is used to estimate model performance. (k-fold cross validation /Bootstrapping /none) | k-fold cross-validation |
|  | Model optimization-cross- N of folds | If k-fold cross validation is used, how many folds are created | 5 |
|  | Model optimization- hyperparameter choosing method | Approaches used to select the best hyperparameter values for the models. (Grid search/ Random search/Domain expert hand search/Bayesian optimization/Other) | Not reported |
|  | Model optimization- Hyperparameter search space reported | Is the searching space of each hyperparameter of the models reported? (It is likely to be reported as a supplementary material) (Yes/No/Not reported) | Not reported |
|  | Model validation type | Type(s) of validation used to examine model performance. (retrospectively internal^2^/ retrospectively external^3/^ prospectively internal/ prospectively external) | retrospectively internal |
|  | Performance metrics | The performance metric(s) used to determine the best model(s) during the development phase and evaluate the model within a testing or validation dataset | AUCROC |
| Results | Model performance-Accuracy | Accuracy of the trained model(s) within a hold-out validation set or external validation set. | Not Reported |
|  | Model performance-AUC/  C-statistics/ AUROC | AUC/C-statistics/AUROC of trained model(s) within a hold-out validation set or external validation set. | 0.81 |
|  | Model performance- Sensitivity | Sensitivity of the trained model(s) within a hold-out validation set or external validation set. | Not Reported |
|  | Model performance-Specificity | Specificity of the trained model(s) within a hold-out validation set or external validation set. | Not Reported |
|  | Model performance-Precision | Precision of the trained model(s) within a hold-out validation set or external validation set. | Not Reported |
|  | Model performance-Recall | Recall of the trained model(s) within a hold-out validation set or external validation set. | Not Reported |
|  | Model performance-NPV | Negative predictive value of the trained model(s) within a hold-out validation set or external validation set. | Not Reported |
|  | Model performance-PPV | Positive predictive value of the trained model(s) within a hold-out validation set or external validation set. | Not Reported |
|  | Model performance-Confusion Matrix reported? | Is confusion matrix of each model reported? | Not Reported |
|  | Model performance-Calibration reported? | Is calibration between model predictions and actual observations reported? | Yes |
|  | Model performance- How well is the model calibration? | How well is the model(s) calibration in general? Good fit/Underfit/overfit/unclear | Good fit |
|  | Model interpretation-Optimal hyperparameter values reported? | Are the best hyperparameter values based on the cross-validation process reported? (Yes/Partially/No) | Yes |
|  | Model interpretation- Important features | Important features (covariates) deemed most important to the model(s) | Not reported |
|  | Model interpretation- Coefficients reported? | Are the Coefficients for the covariates from linear-based regression (such as logistic regression, general linear model, and multivariate adaptive regression splines, etc), if used, reported? (Yes/No) | No |
|  | Model interpretation- decision rules reported? | Are the decision rules developed by tree-based algorithms, if used, (such as decision tree) reported? (Yes/No) |  |
|  | Model interpretation- PDP plot reported? | Is the Partial Dependence Plot for the model(s) used and reported? (Used and Reported/Used but not reported/Not used) | Not used |
|  | Model interpretation- ICE reported? | Is the Individual Conditional Expectation for the model(s) used and reported? (Used and Reported/Used but not reported/Not used) | Not used |
|  | Model interpretation- ALE reported? | Is the Accumulated Local Effects plot for the model(s) used and reported? (Used and Reported/Used but not reported/Not used) | Not used |
|  | Model interpretation- SHAP value reported? | Are the SHapley Additive exPlanations values used and reported? (Used and Reported/Used but not reported/Not used) | Not used |
|  | Model interpretation- LIME reported? | Is the Local Interpretable Model-agnostic Explanations used and reported? (Used and Reported/Used but not reported/Not used) | Not used |
|  | Other interpretation methods | Any other model interpretation methods used |  |
| Conclusion | Model use | Do the authors recommend use of their model? (more research, clinical routine, no use) |  |
|  | Others | Other information that may be useful when writing our paper | None |

**Reference**

1. Oh SE, Seo SW, Choi MG, Sohn TS, Bae JM, Kim S. Prediction of overall survival and novel classification of patients with gastric cancer using the survival recurrent network. Ann Surg Oncol 2018;25(5):1153–1159. PMID:29497908
