# Supplementary material for: Machine Learning–Based Short-Term Mortality Prediction Models for Patients With Cancer Using Electronic Health Record Data: Systematic Review and Critical Appraisal
Source: JMIR Med Inform. 2022 Mar 14;10(3):e33182. doi: 10.2196/33182 (PMC8961346; doi:10.2196/33182)
Supplement: Multimedia Appendix 4 [file medinform_v10i3e33182_app4.docx]

**Multimedia Appendix 4**


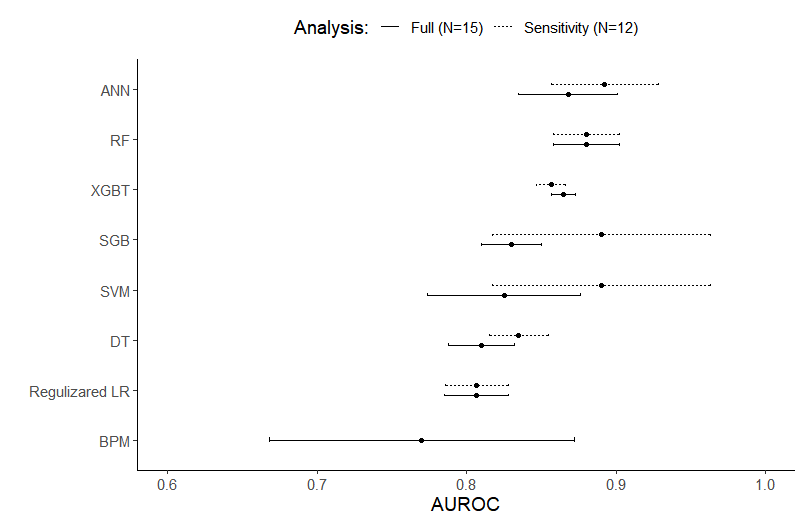


Supplementary Figure 1. Comparison of area under the receiver operating characteristic curve for each machine learning algorithm between the full and sensitivity analyses.

Note:

1. AUROC: area under the receiver operating characteristic curve ANN: artificial neural networks; BPM: Bayes point machine; DT: decision tree; LR: logistic regression; SGB: stochastic gradient boosting; GBT: gradient boosted trees.
2. We included one article per research group in the sensitivity analysis, and thus Manz et al.[1] and Karhade et al.[2,3] were excluded.
3. Differences in AUROC between the analyses ranged from -0.008 to 0.065, with the SVM and SBG having the greatest changes (0.06 and 0.065, respectively).
4. Bayes point machine was only examined in Karhade et al. (2019), and there was no data in the sensitivity analysis for the comparison.

**Reference**

1. Manz CR, Chen J, Liu M, Chivers C, Regli SH, Braun J, Draugelis M, Hanson CW, Shulman LN, Schuchter LM, O’Connor N, Bekelman JE, Patel MS, Parikh RB. Validation of a machine learning algorithm to predict 180-day mortality for outpatients with cancer. JAMA Oncol 2020;6(11):1723–1730. PMID:32970131

2. Karhade A V, Thio QCBS, Ogink PT, Shah AA, Bono CM, Oh KS, Saylor PJ, Schoenfeld AJ, Shin JH, Harris MB, Schwab JH. Development of machine learning algorithms for prediction of 30-day mortality after surgery for spinal metastasis. Neurosurgery 2018;85(1):E83–E91. PMID:30476188

3. Karhade A V, Ahmed AK, Pennington Z, Chara A, Schilling A, Thio QCBS, Ogink PT, Sciubba DM, Schwab JH. External validation of the SORG 90-day and 1-year machine learning algorithms for survival in spinal metastatic disease. Spine J 2020;20(1):14–21. [doi: 10.1016/j.spinee.2019.09.003]
